# Coffee Consumption and Incidence of Subarachnoid Hemorrhage: The Jichi Medical School Cohort Study

**DOI:** 10.2188/jea.JE20150092

**Published:** 2016-02-05

**Authors:** Tsuyako Sakamaki, Motohiko Hara, Kazunori Kayaba, Kazuhiko Kotani, Shizukiyo Ishikawa

**Affiliations:** 1 Graduate School of Saitama Prefectural University, Koshigaya, Saitama, Japan; 1 埼玉県立大学大学院; 2 Department of Clinical Laboratory Medicine, Department of Public Health, Jichi Medical University, Shimotsuke, Tochigi, Japan; 2 自治医科大学臨床検査医学・公衆衛生学; 3 Division of Community and Family Medicine, Center for Community Medicine, Jichi Medical University and The Jichi Medical School Cohort Study Group, Shimotsuke, Tochigi, Japan; 3 自治医科大学地域医療学

**Keywords:** coffee consumption, subarachnoid hemorrhage, community-based cohort study, コーヒー摂取, くも膜下出血, 地域住民を対象としたコホート研究

## Abstract

**Background:**

Previous studies on the association between coffee consumption and subarachnoid hemorrhage (SAH) have provided inconsistent results. We examine the risk of SAH from coffee consumption in a Japanese population.

**Methods:**

Our analyses were based on the Jichi Medical School Cohort Study, a large-scale population-based prospective cohort study. A total of 9941 participants (3868 men and 6073 women; mean age 55 years) with no history of cardiovascular disease or carcinoma were examined. Participants were asked to choose one of five options to indicate their daily coffee consumption: none, less than 1 cup a day, 1–2 cups a day, 3–4 cups a day, or 5 or more cups a day. The incidence of SAH was assessed independently by a diagnostic committee. Cox proportional hazards models were used to calculate hazard ratios (HRs) and their 95% confidence intervals (CI) after adjustment for age and sex (HR1) and for additional potential confounders (HR2).

**Results:**

During 10.7 years of follow-up, SAH occurred in 47 participants. When compared with the participants who consumed less than 1 cup of coffee a day, the HR of SAH was significantly higher in the group who consumed 5 or more cups a day in both models (HR1 4.49; 95% CI, 1.44–14.00; HR2 3.79; 95% CI, 1.19–12.05).

**Conclusions:**

The present community-based cohort study showed that heavy coffee consumption was associated with an increased incidence of SAH after adjusting for age, sex, and multiple potential cardiovascular confounders.

## Abstract (ja)

**背景：:**

コーヒー摂取とくも膜下出血の罹患との関係をみた従来の研究では、一致した結論は得られていない。本研究では地域在住の日本人を対象として、コーヒー摂取とくも膜下出血のリスクの関係について検討した。

**方法：:**

自治医科大学コホート研究の参加者で、循環器疾患や癌の既往のない9,941人（男性：3,868人、女性：6,073人）を対象に、前向きコホート研究を実施した。1日に摂取するコーヒー摂取頻度を、（1）飲まない、（2）1日に1杯未満、（3）1日に1-2杯、（4）1日に3－4杯、（5）1日に5杯以上の5つに区分した。くも膜下出血の罹患は、独立した判定委員会にて判定した。コックスの比例ハザードモデルを用い、ハザード比(HR)とその95％信頼区間(CI)を、交絡因子を調整し（HR1：性と年齢、HR2：性・年齢とその他の交絡因子）算出した。

**結果：:**

追跡期間（平均10.7年）中に、47人がくも膜下出血を発症した。コーヒーを1日に1杯未満摂取と答えた集団に比べて、日に5杯以上摂取すると答えた集団は有意に高い罹患リスクを示した（HR1=4.49, 95%CI:1.44-14.00, HR2=3.79, 1.19-12.05）。

**結果：:**

わが国の地域住民を対象とした本研究では、コーヒーを5杯以上摂取するような大量摂取群でくも膜下出血の罹患が有意に高くなることが示された。

## INTRODUCTION

Coffee is one of the most widely consumed beverages in the world,^1^ although its effects on health are controversial.^2^ As caffeine in coffee elevates blood pressure, coffee drinking has been thought to be a risk factor for incidence of cardiovascular diseases (CVD).^2^ However, some epidemiological studies have reported that coffee intake decreases the risk of cardiovascular^3^^,^^4^ and cerebrovascular diseases.^5^^–^^7^

Subarachnoid hemorrhage (SAH) is a type of severe intracranial bleeding that has a fatality rate of almost 50%.^8^^,^^9^ About 10% of patients die in the prehospital period, and survivors often suffer long-term neurological or cognitive impairments.^8^^–^^10^ Thus, clarifying the risk factors for SAH remains crucial. To date, high blood pressure,^11^^–^^13^ smoking,^11^^–^^15^ and alcohol drinking^15^ have been shown to increase the risk of SAH, while a high body mass index (BMI) decreases the risk.^13^^,^^15^

Given the cerebrovascular effects of coffee,^5^^–^^7^^,^^16^ studies on incident SAH in relation to coffee consumption are important. However, little information on the association between coffee consumption and SAH is available, and results are mixed.^5^^,^^6^^,^^11^^,^^12^ Japan has a nontraditional culture of coffee consumption, with few high-volume consumers of this beverage.^17^ Only one Japanese study has assessed the association of coffee consumption with incident SAH, and the study reported no association.^16^ With the coffee culture in Japan growing and given the mixed results of previous studies, further Japanese research on this topic would be valuable. The purpose of this study was to evaluate the association of coffee consumption in a Japanese population with the incidence of SAH using data from the Jichi Medical School Cohort Study, a large-scale population-based prospective cohort study.^18^

## METHODS

### Subjects

This study used the data of the Jichi Medical School Cohort Study, which enrolled 12 490 participants (4911 men and 7579 women) from 12 communities in Japan.^18^ The Japanese government has conducted mass screening for CVD since 1982 according to a system established by the Health and Medical Service Law for the Aged. The baseline data of the study were obtained during mass screening examinations. The baseline examinations occurred from April 1992 through July 1995, and the examinations included physical examinations, blood tests, and a self-administered questionnaire about socio-demographic status, history of medication use, and diet, including coffee consumption.

Of all participants, 95 declined to participate in follow-up and seven could not be contacted after the baseline examinations. In total, 4869 men and 7519 women were followed up as a complete cohort population. Subjects with a history of CVD or malignant neoplasms and those with missing data on coffee intake were excluded. Ultimately, the data from 9941 participants (3868 men and 6073 women) were used for this study. Further details of the baseline examinations and follow-up methods have been published elsewhere.^18^

### Baseline examination

#### Dietary habits

Dietary habits were assessed using a food frequency questionnaire (FFQ) with 30 items, including an item regarding coffee consumption. Subjects chose one of five options indicating their daily coffee consumption: none, less than 1 cup a day, 1–2 cups a day, 3–4 cups a day, or 5 or more cups a day. The FFQ was already used in the Japan Collaborative Cohort Study, conforming the validity and reproducibility of the frequency assessment.^19^ In order to test the reproducibility, the FFQs were distributed twice, at one-year intervals, and validity was assessed using a weighted dietary record.^19^

#### Lifestyle exposures

The other lifestyle- and health-related exposures were self-reported in semi-structured interviews.^18^ Smoking status was classified as never smoker, ex-smoker, or current smoker. Alcohol consumption was categorized as never drinker, ex-drinker, or current drinker.

#### Physical and blood examinations

Body height was measured without shoes, and weight measured while fully clothed was determined by subtracting 0.5 kg (in the summer) and 1 kg (in other seasons) from the recorded weight values. BMI was calculated as weight in kilograms divided by squared body height in meters. Systolic blood pressure (SBP) was measured using an automated sphygmomanometer on the right arm of the participants after sitting for 5 minutes. Serum concentrations of total cholesterol (TC), triglyceride (TG), and high-density lipoprotein (HDL) cholesterol were measured using an enzymatic method.

### Follow-up

The health status of the participants was followed up each year after the baseline examination. Participants were asked whether they had a diagnosis of CVD and, if so, which hospital they had visited and when they received the diagnosis. Additionally, if the participants did not attend the screening examination each year, they were contacted by mail, telephone, or via a public health nurse’s home visit to obtain information on their health status. Death certificates of the participants were collected from public health centers with permission from the Agency of General Affairs and the Ministry of Health, Labour and Welfare. The follow-up of participants who died before the end of the study was stopped at that time. Information on participants who moved out of the study communities during the follow-up period was obtained annually from the relevant municipal governments; these participants (*n* = 340) were no longer followed up from the day they left the study communities. Follow-up of all other participants was continued until the end of 2005.

### Diagnostic criteria of CVD, including SAH

In this study, CVD was defined as stroke, myocardial infarction, and sudden cardiac death, whichever occurred first. In participants with an event suspected to be related to CVD, computed tomography (CT) scans or magnetic resonance images in cases of stroke or electrocardiograms in cases of myocardial infarction was duplicated. A set of the image copy was sent to the diagnostic committee. CVD events were diagnosed independently by a diagnosis committee, which was composed of a neurologist, a radiologist, and two cardiologists. Stroke was diagnosed according to the diagnostic criteria of the National Institute of Neurological Disorders and Stroke (ie, in cases with a sudden onset of a focal, non-convulsive, and neurological deficit persisting longer than 24 hours).^20^ SAH was diagnosed with a cranial CT scan performed to confirm the hyperdense appearance of extravasated blood in the subarachnoid space and/or basal cisterns. Myocardial infarction was diagnosed according to the criteria of the World Health Organization Multinational Monitoring of the Trends and Determinants in Cardiovascular Disease (MONICA) Project.^21^

### Statistical analysis

The Statistical Package for Social Science (SPSS) for Windows, version 21.0 (IBM SPSS Japan Inc., Tokyo, Japan) was used for all analyses. General characteristics of participants were analyzed by frequency of coffee consumption and reported as proportions and means (standard deviations). The associations between the frequency of coffee consumption and the confounders were analyzed by one-way analysis of variance and the chi-square test. A Cox proportional hazards model was used for calculating the hazard ratios (HRs) and 95% confidence intervals (CIs) of the incidence of SAH in relation to categories of coffee consumption, with adjustment for age and sex (HR1) or adjustment for age, sex, BMI, SBP, TC, smoking status, and alcohol consumption (HR2). Age, BMI, SBP, and TC were entered in the model as continuous variables; sex, smoking (current, ex-, or never smoker), and alcohol drinking (current, ex-, or never drinker) were entered as categorical variables.

### Ethical considerations

This study was approved by the Institutional Review Board of Jichi Medical School (Epidemiology 03-01) and the Ethics Committee of Saitama Prefectural University (524716). Written informed consent was obtained from each participant.

## RESULTS

The baseline characteristics by frequency of coffee intake are shown in Table 1. High-frequency drinkers were more likely to be young, smokers, and alcohol drinkers and less likely to be female and obese. The group who drank 3–4 cups of coffee a day had lower SBP and TG.

**Table 1.** Baseline characteristics of participants by frequency of coffee intake

	Frequency of coffee intake						

	None	Less than 1 cup a day	1–2 cups a day	3–4 cups a day	5 or more cups a day	Total	*P*-value^a^
Number of subjects	2631	3198	2924	883	305	9941	
Female, %	65.1	60.6	60.9	56.9	44.6	61.1	<0.001
Age, years	59.8	56.1	51.2	48.1	52.3	54.8	<0.001
	(9.8)	(10.8)	(11.9)	(12.2)	(12.5)	(11.7)	
Body mass index, kg/m^2^	23.1	23.1	23.0	22.8	22.8	23.0	0.011
	(3.2)	(3.0)	(3.0)	(3.0)	(3.1)	(3.1)	
Systolic blood pressure, mm Hg	132.2	129.9	126.3	123.6	126.1	128.8	<0.001
	(20.9)	(20.7)	(20.6)	(19.8)	(21.7)	(20.9)	
**Serum cholesterol concentration**							
Total cholesterol, mg/dL	192.5	191.7	191.1	189.5	188.1	191.5	0.078
	(34.5)	(34.8)	(35.4)	(35.3)	(32.4)	(34.9)	
HDL cholesterol, mg/dL	51.0	50.9	51.3	51.1	49.4	51.0	0.150
	(12.9)	(12.7)	(12.8)	(12.9)	(13.6)	(12.8)	
Triglycerides, mg/dL	122.0	118.1	110.0	109.3	117.4	115.9	<0.001
	(77.2)	(74.1)	(71.7)	(77.1)	(74.4)	(74.7)	
Current smoker, %	16.4	20.2	26.2	38.5	50.5	23.5	<0.001
Current alcohol drinker, %	36.4	44.0	48.2	54.8	54.2	44.5	<0.001

HDL, high-density lipoprotein.

^a^Values were calculated using one-way analysis of variance for continuous variables or the chi-square test for categorical variables and are reported as mean (standard deviation) unless otherwise noted.

During an average follow-up of 10.7 years, we documented 488 CVD events (270 in men and 218 in women): 360 strokes (187 in men and 173 in women) including 47 SAHs (13 in men and 34 in women), 84 hemorrhagic strokes (42 in men and 42 in women), and 228 cerebral infarctions (132 in men and 96 in women). The incidence of SAH was 4.4 per 10 000 person-years.

Adjusted HRs and 95% CIs by frequency of coffee intake are shown in Table 2. HRs of SAH incidence were significantly higher among those who drank 5 or more cups a day than in those who drank less than 1 cup a day (HR1 4.49; 95% CI, 1.44–14.00 and HR2 3.79; 95% CI, 1.19–12.05).

**Table 2.** Hazard ratios and 95% confidence intervals for the incidence of subarachnoid hemorrhage by frequency of coffee intake adjusted for potential cardiovascular confounders

	Frequency of coffee intake				

	None	Less than 1 cup a day	1–2 cups a day	3–4 cups a day	5 or more cups a day
Person-years	27 719	34 682	31 629	9442	3200
Number of cases					
Total	15	12	13	3	4
Men	5	1	4	2	1
Women	10	11	9	1	3
Incidence rate, per 10 000 person-years	5.4	3.5	4.1	3.2	12.5
HR1 (95% CI)	1.29 (0.60–2.77)	1.00	1.44 (0.65–3.17)	1.28 (0.36–4.60)	4.49 (1.44–14.00)
HR2 (95% CI)	1.31 (0.61–2.82)	1.00	1.28 (0.57–2.87)	1.16 (0.32–4.23)	3.79 (1.19–12.05)

CI, confidence interval; HR1, Hazard ratio adjusted for age and sex; HR2: Hazard ratio adjusted for age, sex, body mass index, systolic blood pressure, total cholesterol concentration, smoking status, and alcohol consumption.

## DISCUSSION

The present study found that subjects who consumed 5 or more cups of coffee a day had a significantly higher risk of SAH incidence, while no significant risk increase was observed among those who drank less than 5 cups a day. To our knowledge, this is the first report of a significant increase of SAH incidence among heavy coffee drinkers in Japan. Given the mixed epidemiological research results on the cerebrovascular effects of coffee,^5^^–^^7^^,^^11^^,^^12^^,^^16^ this finding is valuable.

Among our subjects, those who consumed 5 or more cups of coffee a day can be regarded as extremely high consumers. Such individuals might have other unhealthy nutrition-taking behaviors, meaning that the magnitude of the risk could be exaggerated. Further nutritional study is needed.

The Miyagi cohort study in Japan reported that frequent coffee intake was not significantly correlated with SAH mortality.^16^ The Miyagi study categorized the frequency of coffee intake into three groups (never, occasionally, and one or more cups a day) while our analysis used five groups. This different categorization of coffee consumption could explain the different results.

Most previous studies of the association between coffee intake and SAH were conducted in Western countries; two of these were incidence studies. Swedish women with high coffee intake showed significantly lower SAH incidence,^6^ but coffee intake was not significantly associated with SAH incidence among Finnish male smokers.^5^ Subjects in the Swedish study were about 60 years old and were participants in a mammography program. Their measured coffee intake was similar to that of our subjects, but they were older than our subjects by about 5 years on average and had a lower incidence of SAH (2.2 per 10 000 person-years). Compared with the subjects in our study, they may represent a healthier population. Age and incidence of SAH (5.4 per 10 000 person-years) of the Finnish men were similar to those of our subjects, but they were smokers and heavier coffee drinkers, and 21% reported consuming 8 or more cups of coffee a day. These differences in characteristics between the Scandinavian subjects and our own may help explain the differences in the findings.

A Colombian case-control study found no significant association between coffee intake and SAH.^12^ Our results appear to be consistent with those of a Norwegian study^11^ that showed significantly increased SAH mortality among subjects who drank more than 6 cups of coffee a day.

Findings from the present epidemiological study cannot fully explain the underlying mechanisms of the relation between coffee consumption and incidence of SAH. Many experimental and clinical studies have reported both protective and harmful effects of coffee. Excess intake of caffeine, the most investigated component in coffee, may elevate blood pressure by increasing systemic vascular resistance.^16^ Hydroxyhydroquinone generated by roasting coffee beans could interfere with the vasodilatory effect of chlorogenic acids,^2^ which have antioxidant functions^17^ that benefit vascular health.^22^^,^^23^ Another possibility is that the addition of sugar, milk, and cream to coffee leads to high energy intake that may induce oxidative stress and insulin resistance. However, when our study results were adjusted to account for factors related to oxidative stress and insulin resistance, including smoking, BMI, and blood pressure, the adjustments did not attenuate the statistical significance of the findings.

The strengths of this study are the large size, use of a community-based cohort study design with incident disease outcomes, and careful case review by an independent diagnostic committee. However, our study has several limitations. First, the group with a significantly increased risk of SAH included only four cases. While the results were statistically significant after adjusting for sex, age, and five major CVD risk factors, the robustness of the observed association is probably limited, and the finding could be a chance observation. No statistically significant trend in risk was observed among those who drank less than 5 cups a day. Statistical power may be low due to the small number of SAH cases (*n* = 47) in the cohort population, while the incidence of SAH in this study is almost three times as high as the mortality of SAH (1.46 per 10 000 person-years) reported in a previous Japanese study.^16^ Second, a high prehospital mortality rate could make SAH diagnosis difficult. During follow-up, we documented 41 cases of sudden death defined as death within 24 hours after the onset of symptoms. All cases of sudden death were reviewed carefully by the diagnostic committee to rule out SAH. However, considering difficulty in identifying cause of out of hospital death, it is possible that some SAH cases could not be diagnosed. Finally, the FFQ was self-administered and implemented only once at baseline, so the evaluation of dietary habits might not be accurate. We did not clarify whether dietary habits changed during the follow-up period, although the validity and reliability of the FFQ are known to be acceptable.^19^ Types of coffee and its additives were not assessed by the FFQ.

In conclusion, the present study from the Jichi Medical School Cohort Study showed that, compared with subjects who consumed less than 1 cup a day, those who consumed 5 or more cups of coffee a day had a significantly higher risk of incident SAH, while no significant increase in risk was observed among those who drank less than 5 cups a day. This suggests that heavy coffee consumption is a risk factor for incident SAH.

## ONLINE ONLY MATERIALS

Abstract in Japanese.
