# Supplementary material for: Coffee Consumption and Incidence of Subarachnoid Hemorrhage: The Jichi Medical School Cohort Study
Source: J Epidemiol. 2016 Feb 5;26(2):71–5. doi: 10.2188/jea.JE20150092 (PMC4728117; doi:10.2188/jea.JE20150092)
Supplement: Abstract in Japanese. [file je-26-071-s001.pdf]

コーヒー摂取とくも膜下出血の罹患：自治医科大学コホート研究

坂巻つや子[1]、原元彦[1]、萱場一則[1]、小谷和彦[2]、石川鎮清[3]

[1]埼玉県立大学大学院、[2]自治医科大学臨床検査医学・公衆衛生学、[3]自治医科大学地域医療学

背景：コーヒー摂取とくも膜下出血の罹患との関係をみた従来の研究では、一致した結論は得られていない。本研究では地域在住の日本人を対象として、コーヒー摂取とくも膜下出血のリスクの関係について検討した。

方法：自治医科大学コホート研究の参加者で、循環器疾患や癌の既往のない 9,941 人（男性：3,868 人、女性：6,073 人）を対象に、前向きコホート研究を実施した。1 日に摂取するコーヒー摂取頻度を、(1) 飲まない、(2) 1 日に 1 杯未満、(3) 1 日に 1-2 杯、(4) 1 日に 3－4 杯、(5) 1 日に 5 杯以上の 5 つに区分した。くも膜下出血の罹患は、独立した判定委員会にて判定した。コックスの比例ハザードモデルを用い、ハザード比(HR)とその 95%信頼区間(CI)を、交絡因子を調整し（HR1：性と年齢、HR2：性・年齢とその他の交絡因子）算出した。

結果：追跡期間（平均 10.7 年）中に、47 人がくも膜下出血を発症した。コーヒーを 1 日に 1 杯未満摂取と答えた集団に比べて、日に 5 杯以上摂取すると答えた集団は有意に高い罹患リスクを示した（HR1=4.49, 95%CI:1.44-14.00, HR2=3.79, 1.19-12.05）。

結果：わが国の地域住民を対象とした本研究では、コーヒーを 5 杯以上摂取するような大量摂取群でくも膜下出血の罹患が有意に高くなることが示された。

キーワード：コーヒー摂取、くも膜下出血、地域住民を対象としたコホート研究
